# Salvage radiotherapy after radical prostatectomy: analysis of toxicity by dose-fractionation in the RADICALS-RT trial

**DOI:** 10.1016/j.ijrobp.2023.04.032

**Published:** 2023-05-06

**Authors:** Peter Meidahl Petersen, Adrian D Cook, Matthew R Sydes, Noel Clarke, William Cross, Howard Kynaston, John Logue, Peter Neville, Heather Payne, Mahesh KB Parmar, Wendy Parulekar, Rajendra Persad, Fred Saad, Alan Stirling, Christopher C Parker, Charles Catton

**Affiliations:** 1 Department of Oncology, Rigshospitalet, Copenhagen University Hospital, Copenhagen, Denmark; 2 MRC Clinical Trials Unit at UCL, Institute of Clinical Trials and Methodology, UCL, London, UK; 3 The Christie and Salford Royal Hospitals, Manchester University, Manchester, UK; 4 Department of Urology, St James's University Hospital, Leeds UK; 5 Howard Kynaston, MD, Professor. University Hospital of Wales, Cardiff, UK; 6 The Christie NHS Foundation Trust, Manchester, UK; 7 University College London Hospitals, The Prostate Centre, London, UK; 8 Canadian Cancer Trials Group, Queen’s University, Kingston, Canada, CA; 9 Bristol Urological Institute, North Bristol Hospital, Bristol, UK; 10 Fred Saad MD, Professor. Urologic Oncology, Centre Hospitalier de l’Université de Montréal. Montreal, Canada; 11 Castle Hill Hospital, Castle Road, Cottingham, UK; 12 The Institute of Cancer Research, Royal Marsden NHS, Foundation Trust, Sutton, UK; 13 Radiation Oncology, Princess Margaret Hospital, University Health Network, MGM - Toronto, CA

## Abstract

Emerging data indicate comparable disease control and toxicity of postoperative normo-fractionation and moderate hypofractionation radiotherapy (RT) in prostate cancer. In RADICALS-RT, patients were planned for treatment with either 66Gy in 33 fractions over 6.5 weeks or 52.5Gy in 20 fractions over 4 weeks. In this non-randomized, exploratory analysis, we explore the toxicity of these two schedules in patients who had adjuvant RT.

**Methods:**

Information on RT dose was collected in all patients. Radiation Therapy Oncology Group toxicity score was recorded every 4 months for 2 years, 6-monthly until 5 years, then annually until 15 years. Patient-reported data were collected at baseline, 1, 5, and 10 years with use of standard questionnaires including Vaizey (bowel) and International Continence Society Male Short-Form (urinary incontinence). The highest grade of event was recorded within the first 2 years, and beyond 2 years, and compared between treatment groups using the χ^2^ test.

**Results:**

217/634 (34%) patients were planned for 52.5Gy/20f and 417/634 (66%) for 66Gy/33f. In the first two years, grade 1 – 2 cystitis was reported more frequently among the 66Gy/33f group (52.5Gy/20f: 20% vs 66Gy/33f: 30%, p=0.04). After two years, grade 1-2 cystitis was reported in 16% in the 66Gy group, and 9% in the 52.5Gy group (p=0.08). Other toxicities were similar in the two groups and very few patients had any grade 3 – 4 toxicity.

Patients reported slightly higher urinary and faecal incontinence scores at one year than at baseline, but no clinically meaningful differences were reported between 52.5Gy/20f and 66Gy/33f groups. Patient reported health was similar at baseline and at one year, and similar between 52.5Gy/20f and 66Gy/33f groups.

**Conclusion:**

Severe toxicity is rare after prostate bed radiotherapy with either 52.5Gy/20f or 66Gy/33f. Only modest differences were recorded in toxicity or in patient reported outcomes between these two schedules.

## Introduction

The aim of the RADICALS-RT trial was to test, in men having surgery for prostate cancer, the efficacy and safety of adjuvant radiotherapy versus a policy of observation with early salvage radiotherapy for PSA progression. The early results did not support the use of adjuvant RT after radical prostatectomy ^1^.

In primary RT for low-and intermediate-risk prostate cancer, moderately-hypofractionated RT schedules have comparable tumor control and toxicity with normo-fractionated RT^2–4^. Phase II studies have shown acceptable toxicity in patients treated with hypofractionated radiation after prostatectomy ^5,6^. Recent data in post-prostatectomy RT have also indicated comparable short-term toxicity of normo-fractionation and moderate hypofractionation ^7^.

In RADICALS-RT, there was a choice of one of two dose-fractionation schedules for prostate bed radiotherapy: 66Gy in 33 fractions over 6.5 weeks (“66Gy/33f group”) or 52.5Gy in 20 fractions over 4 weeks (“52.5Gy/20f group”). This choice was made on an individual patient basis, nominated prior to randomization and was used as a stratification variable. Here we explore the toxicity of 52.5Gy/20f compared to 66Gy/33f in patients who had adjuvant RT in the RADICALS-RT trial.

## Methods

RADICALS is an international, phase III, multi-centre, open-label, randomised controlled trial for men with prostate cancer. Participants were randomly assigned shortly after radical prostatectomy, where there was uncertainty about RT use, to adjuvant or salvage postoperative radiotherapy (RADICALS-RT) and, in participants planned for postoperative radiotherapy, to add short-course (6 months), long-course (24 months) or no hormone therapy (RADICALS-HD). Details of the protocol have been published previously ^1^.

RT was planned according to the choice of the treating physician as either 52.5Gy/20f or 66Gy/33f. Hence, the choice of RT schedule was not randomised. Treatment with either 3-D conformal therapy or with intensity modulated radiotherapy (IMRT) was allowed. The treatment was targeted to the prostate bed or prostate bed and pelvic lymph nodes as decided by the treating physician. The protocol included a guideline on contouring and treatment planning and each participating center went through an accreditating process. As per protocol, the minimal dose to the PTV was 95% of and the maximum dose 105% of the prescribed dose.

Patients were seen every 4 months for 2 years, 6-monthly until 5 years, then annually until 15 years. Radiation Therapy Oncology Group (RTOG) toxicity scores were collected at each follow-up visit regarding diarrhoea, proctitis, cystitis, haematuria, and urethral stricture. Data classified as a serious adverse event were also collected. Patient-reported data were collected at baseline, 1, 5, and 10 years post-randomisation with use of standard questionnaires that included Vaizey (bowel) and International Continence Society Male Short-Form (urinary incontinence).

Toxicity data were dichotomized into events reported within 2 years after randomisation and after more than 2 years. Within each period the highest grade of event was compared between groups using the χ^2^ test. For patient-reported outcomes, groups were compared at 1 and 5 years using analysis of covariance adjusted for baseline score. Stata, version 16.1 was used for statistical analysis. No adjustment was made for baseline characteristics such as use of hormone therapy or radiotherapy technique. This analysis uses the dataset used for the early reporting of biochemical outcomes, frozen on 21 March 2019 ^1^.

## Results

### Patient characteristics

217/634 (34%) of the participants receiving adjuvant RT were planned for 52.5Gy/20f and 417/634 (66%) for 66Gy/33f (Table 1). Median follow-up was 4.9 years IQR(3.1,6.1) in these 634 patients. No difference was seen regarding age between the two groups. However, less patients with 52.5Gy/20f planned had pT3b/pT4 disease than those planned for 66Gy (13% (n=28/217) vs 21% (n=90/417), p=0.008) and fewer had a Gleason score of 8-10 (11% (n=24/217) vs 19% (n=79/417), p=0.001). Patients with 66Gy/33f planned were significantly more likely to have RT targeted at the pelvic nodes as well as the prostate bed (7% vs 1%, p=0.002) (Table 2). There was no difference between groups in the size of the prostate bed target volume of radiotherapy and almost all patients received their planned schedule, starting treatment within the protocol-defined time windows. Most patients (95% in both groups) received their intended dose.

### Toxicity

Toxicity data are shown in Table 3. In the first two years grade 1–2 cystitis was reported more frequently for the 66Gy/33f group (52.5Gy/20f: 20% vs 66Gy/33f: 30%, p=0.02). Beyond two years grade 1-2 cystitis was reported for 16% among the 66Gy/33f group and 9% for the 52.5Gy/20f group (p=0.08). Other toxicities were similar for both 52.5Gy/20f and 66Gy/33f patients.Grade 3–4 toxicity was reported in very few patients.

### Quality of-life

Urinary and faecal incontinence scores were slightly higher one year after randomisation than at baseline but the change was not clinically meaningful. The differences between 52.5Gy/20f and 66Gy/33f groups were not statistically significant (Table 4). Overall, patients reported similar physical and mental health scores at baseline and at one year and these were similar between the 52.5Gy/20f and 66Gy/33f groups.

## Discussion

These exploratory, non-randomised data from the adjuvant RT group of RADICALS-RT indicate that prostate bed radiotherapy following radical prostatectomy was usually well tolerated regardless of the choice of dose fractionation schedule. The shorter 20 fraction schedule offers an obvious advantage in terms of patient convenience, environmental considerations and hospital capacity.

This current analysis is not designed to test differences in disease outcome between the two dose-fractionation groups. However, 64Gy/32 fractions seems to be sufficient for biochemical control in the absence of macroscopic local recurrence in the prostate bed ^8^, a dose biologically equivalent to the 52,5Gy/20f schedule, so it is not expected that there will be a difference in efficacy by choice of fractionation schedule in RADICALS-RT.

The treatment time was shorter in the 52.5Gy/20f group. The impact of the shorter treatment time on toxicity is uncertain but might be expected, if anything, to increase acute toxicity ^9,10^: This phenomenon was not seen in this study. In RADICALS only a minority had pelvic node radiation, with more of these in the 66 Gy/33f than the 52.5Gy/20f group (7% vs 1%). This fact could bias our results to an extent as pelvic RT could increase GI toxicity. However, other studies did not show increased late bowel toxicity with pelvic node RT ^11,12^.

One retrospective study has suggested increased late urinary toxicity with moderate hypofrationation compared to normo-fractionation^13^. The late urinary toxicity in RADICALS was comparable to the toxicity in the normo-fractionated patients in the previous study. This difference could be explained by the relatively high dose level corresponding to 2 Gy dose schedules (BED calculated using an α/β-ratio of 5 for urohtel) in the previous study^13^.

Interaction between hormone treatment and GI toxicity has been suggested previously (10,11). As we did not see any difference in use of ADT between the 2 groups this potential interaction did not bias the results of the present analysis.This exploratory analysis has limitations. First, the comparison between the two dose-fractionation schedules was non-randomised. Second, radiotherapy techniques have continued to evolve since RADICALS-RT recruitment between 2008 and 2016. Subsequent radiotherapy techniques would be expected to further reduce dose to normal tissues such as rectum and bladder, and thus further reduce the risk of radiation toxicity. Third, post-operative radiotherapy to the prostate bed is now more commonly delivered in the salvage setting rather than the adjuvant setting following publication of the results of the RADICALS-RT, GETUG-17, RAVES trials and the ARTISTIC meta-analysis ^1,14–16^. However, it is reasonable to assume that the toxicity of post-operative radiotherapy would be similar in the adjuvant and in the salvage setting. Fourth, the choice of treatment schedule was closely associated with centre. We cannot completely rule out that this association has an impact on the toxicity outcomes. The strengths of our analysis include the prospective collection of clinician - reported and patient-reported data in a pre-defined population in the context of a clinical trial.

## Conclusion

Severe toxicity is rare after post prostatectomy prostate bed radiotherapy with either 52.5Gy/20f or with 66Gy/33f. Only modest differences were seen in toxicity or in patient reported quality of life between these two schedules. In the interests of patient convenience, hospital capacity, and environmental considerations, these exploratory results support the use of hypofractionated radiotherapy to the prostate bed.

## Figures and Tables

**Table 1:** Patient Characteristics, n (%) unless indicated

	52.5 Gy		66 Gy		
	N	%	N	%	p
	217	(100)	417	(100)	
**Age**					
Years*	64 (60,68)		65 (59,68)		0.86
**PSA at diagnosis**					
ng/ml*	7.5 (5.6,11)		7.9 (6,12.1)		0.047
**Gleason score**					
GS <7	18	(8)	23	(6)	
GS 3+4	129	(59)	192	(46)	
GS 4+3	46	(21)	123	(30)	
GS ≥8	24	(11)	79	(19)	0.001
**Pathologic T stage**					
pT2	43	(20)	99	(24)	
pT3a	146	(67)	228	(55)	
pT3b	28	(13)	85	(20)	
pT4	0	(0)	5	(1)	0.008
**Positive margins**					
Present	142	(65)	258	(62)	
Absent	75	(35)	159	(38)	0.38
**Lymph node involvement**					
N1	5	(2)	28	(7)	
N0	115	(53)	196	(47)	
Nx	96	(44)	192	(46)	0.039
**CAPRA-S score**					
Low (0 to 2)	21	(10)	31	(7)	
Intermediate (3 to 5)	133	(61)	214	(51)	
High (6+)	63	(29)	172	(41)	0.010
**Country**					
England	217	(100)	321	(77)	
Denmark	0	(0)	74	(18)	
Canada	0	(0)	19	(5)	
Republic of Ireland	0	(0)	3	in	<0.001

* median (IQR)

**Table 2:** Radiotherapy treatment

		52.5 Gy		66 Gy		
		N	%	N	%	p
		217	(100)	417	(100)	
**Planned RT target**						
Prostate bed			214 (99)		388 (93)	
Prostate bed and pelvic lymph nodes			3 (1)		29 (7)	0.002
Target volume (PTV_prostate bed)						
cc^3^*			280		277	0.52
			(228,332)		(218,354)	
**Dose given (Gy)**						
Median	p50		52.5		66	
IQR	p25, p75		52.5, 52.6		66, 66	
	p10, p90		52.4, 54.2		66, 66	
Range	p0, p100		2.6, 66		20, 68	
**Randomisation to RT (days)**						
**Not starting HT**			n=174		n=328	
Median (IQR)		34	(29,47)	36	(28,56)	0.17
**Starting HT**			n=43		n=89	
Median (IQR)		74	(62,88)	74	(68,90)	0.10

* median (IQR)

**Table 3:** RTOG toxicity

	Within 2 years						After 2 years			
	52.5Gy		66Gy			52.5Gy		66Gy		
	N	%	N	%	p	N	%	N	%	p
	217	(100)	418	(100)		195	(100)	371	(100)	
**Diarrhoea**										
Grade 1	64	(29)	130	(31)	0.10	24	(12)	57	(15)	0.48
Grade 2	26	(12)	36	(9)		6	(3)	16	(4)	
Grade 3	6	(3)	3	(1)		2	(1)	3	(1)	
Grade 4	0	(0)	0	(0)		1	(1)	0	(0)	
**Proctitis**										
Grade 1	35	(16)	64	(15)	0.77	21	(11)	32	(9)	0.46
Grade 2	12	(6)	32	(8)		9	(5)	11	(3)	
Grade 3	2	(1)	5	(1)		1	(1)	5	(1)	
Grade 4	0	(0)	0	(0)		0	(0)	0	(0)	
**Cystitis**										
Grade 1	30	(14)	87	(21)	0.04	10	(5)	40	(11)	0.08
Grade 2	12	(6)	36	(9)		7	(4)	20	(5)	
Grade 3	2	(1)	9	(2)		1	(1)	4	(1)	
Grade 4	0	(0)	1	(<1)		0	(0)	0	(0)	
**Haematuria**										
Grade 1	20	(9)	28	(7)	0.73	11	(6)	23	(6)	0.87
Grade 2	7	(3)	14	(3)		13	(7)	22	(6)	
Grade 3	7	(3)	13	(3)		6	(3)	16	(4)	
Grade 4	0	(0)	0	(0)		0	(0)	1	(<1)	
**Urethral stricture**										
Grade 1	8	(4)	14	(3)	0.58	8	(4)	16	(4)	0.33
Grade 2	7	(3)	12	(3)		3	(2)	9	(2)	
Grade 3	9	(4)	28	(7)		5	(3)	21	(6)	
Grade 4	0	(0)	2	(<1)		0	(0)	0	(0)	

**Table 4:** Quality of life 1 year after randomisation

	52.5Gy		66Gy		
	N	%	N	%	p
	217	(100)	417	(100)	
**ICS male incontinence score**					
Data at baseline and 1 year	135	(62)	207	(50)	
Baseline score, mean (sd)	4.39	(3.79)	4.71	(3.72)	
1 year score, mean (sd)	4.42	(3.92)	5.05	(3.99)	
Difference between arms at 1 year*					
Mean (95% CI)		0.34	(-0.37,1.05)		0.35
**Vaizey faecal incontinence score**					
Data at baseline and 1 year	119	(55)	196	(47)	
Baseline score, mean (sd)	2.45	(3.23)	2.51	(3.32)	
1 year score, mean (sd)	3.27	(3.61)	3.37	(4.02)	
Difference between arms at 1 year*					
Mean (95% CI)		0.07	(-0.74,0.88)		0.86
**SF-12 physical health score** ^†^					
Data at baseline and 1 year	123	(57)	193	(46)	
Baseline score, mean (sd)	51.3	(7.8)	50.3	(8.5)	
1 year score, mean (sd)	51.5	(7.6)	49.9	(9.2)	
Difference between arms at 1 year*					
Mean (95% CI)		-1.42	(-3.15,0.32)		0.11
**SF-12 mental health score** ^††^					
Data at baseline and 1 year	123	(57)	193	(46)	
Baseline score, mean (sd)	51.6	(9.6)	51.2	(9.5)	
1 year score, mean (sd)	52.9	(9.2)	52.1	(8.6)	
Difference between arms at 1 year*					
Mean (95% CI)		-0.62	(-2.42,1.18)		0.50

* adjusted for baseline score, age, t-stage and Gleason score

† possible score range 13-69,

†† possible score range 10-70

## Data Availability

The dataset may be available upon request as per the moderated access approach of the MRC Clinical Trials Unit at UCL. Please contact the corresponding author for more information.
